# Bottom‐up citizen initiatives as emergent actors in flood risk management: Mapping roles, relations and limitations

**DOI:** 10.1111/jfr3.12468

**Published:** 2018-08-17

**Authors:** Sebastian Seebauer, Stefan Ortner, Philipp Babcicky, Thomas Thaler

**Affiliations:** ^1^ LIFE – Centre for Climate, Energy and Society Joanneum Research Forschungsgesellschaft mbH Graz Austria; ^2^ Department of Geography University of Innsbruck Innsbruck Austria; ^3^ Wegener Center for Climate and Global Change and FWF‐DK Climate Change University of Graz Graz Austria; ^4^ Institute of Mountain Risk Engineering University of Natural Resources and Life Sciences Vienna Austria

**Keywords:** citizen participation, civic protest, disaster risk management, flood action group, grassroots movement, risk governance

## Abstract

The recent shift to individualisation of flood risk calls for a stronger involvement of private actors. Bottom‐up citizen initiatives (BUIs) may bring together governmental bodies with people at risk. Drawing on a screening of existing BUIs in Europe, North America, and Australia and an in‐depth analysis of three study sites, this paper maps BUI activities to stages in the risk management cycle and discusses the institutional, relational and social proximity between BUIs and other stakeholders. Flood BUIs often take over roles that the authorities are not willing or able to fulfil. BUIs emerge out of frustration with current risk policies, after a catastrophic flood event, government‐initiated engagement projects or targeted funding opportunities. BUIs can take different forms, ranging from oppositional pressure groups, self‐help movements for disaster response and recovery, to initiatives formally installed by law. While self‐organised BUIs benefit from high proximity to their home communities, formalised BUIs are deeper embedded in existing institutional structures. In order to gain a stronger voice in the risk debate, BUIs need to expand from the local level to catchment areas and exchange expertise and resources in nationwide or cross‐border networks. However, BUIs may create parallel political structures that are not democratically legitimised.

## 1 INTRODUCTION

Increasing flood risks under climate and societal change (IPCC, 2014) significantly transform the roles held by different actors in flood risk management. Government authorities struggle to carry the full load of protecting and recovering from extreme hydrological events (Pfurtscheller & Thieken, 2013). Thus, the forthcoming policy agenda strives to delegate responsibilities and costs from the civil authorities to private citizens (Adger, Quinn, Lorenzoni, & Murphy, 2016; Thaler & Priest, 2014). As international strategies such as the Local Agenda 21 (UN Conference on Environment and Development, 1992), the EU Floods Directive (EU, 2007), or the Sendai Framework for Disaster Risk Reduction (UNISDR, 2015) are implemented in national jurisdictions, centralised policy directions are increasingly combined with local strategies for managing local risks with local stakeholders (van Aalst, Cannon, & Burton, 2008; Veraart, van Nieuwaal, Driessen, & Kabat, 2014). Among those local stakeholders, concerned citizens are meant to take centre stage (Thaler & Levin‐Keitel, 2016).

### 1.1 Citizen initiatives in risk governance

Giving citizens at risk a stronger role in flood risk management has already been on the agenda for many decades (e.g., Arnstein, 1969). Against this background, the recent concept of risk governance emphasises stronger citizen engagement (Renn, 2008). Involving citizens is seen as essential for leveraging local risk expertise, legitimising policy decisions, or establishing compliance with emergency procedures (Dilling & Lemos, 2011; Moser & Ekstrom, 2010). To this end, power, responsibility and policy outcomes need to be balanced and co‐produced between professionals and involved citizens (Albrechts, 2013; Boyle & Harris, 2009; Innes & Booher, 2004).

However, citizens rarely enter the risk discourse as individuals, but pool their influence and resources (Kruse & Seidl, 2013; Kuhlicke et al., 2011). The present paper aims to shed light on the emerging role of bottom‐up citizen initiatives (BUIs), understood here as community‐based, participatory groups, in which private households at risk band together for local preventive action, flood response, recovery, or lobbying governmental bodies. Self‐organised BUIs, on the one hand, emerge as local grassroots groups founded by committed activists as a response to the inadequacy and ineffectiveness of mere top‐down policy (van Aalst et al., 2008; Veraart et al., 2014). Formalised BUIs, on the other hand, are initiated by government authorities seeking cooperation or transfer of responsibility from public to private actors (Edelenbos, Van Buuren, Roth, & Winnubst, 2017; Mees, 2013).

In light of the upcoming risk governance agenda, BUIs may bridge the gap between mainstream, centralised institutions and individual citizens (Djordjevic, Butler, Gourbesville, Mark, & Pasche, 2011; Termeer et al., 2011). As intermediaries and facilitators, BUIs may help to align the views of individuals and institutions (Reed et al., 2009; Stanghellini, 2010). BUIs may act as dialogue partners that civil servants are more willing to accept as counterpart than they would free‐floating individuals (Driessen, Dieperink, Laerhoven, Runhaar, & Vermeulen, 2012; Levin‐Keitel, 2014). Nevertheless, government agencies are sometimes reluctant to embrace BUI activity, as open civic dialogue easily turns into protest and media attention that impede rather than support deliberative decision processes (Blackstock et al., 2015; Buchecker, Ogasa, & Maidl, 2016; Newig, Kochskämper, Challies, & Jager, 2016).

### 1.2 Objectives

The aim of this paper is to illustrate how bottom‐up initiatives emerge and establish themselves in local flood risk management. To this end, we seek to answer the following research questions:

1. Which activities are undertaken by BUIs, and how did those activities develop against the background of citizen needs and governmental practice?
2. How do BUIs relate to other actors in the risk governance arena, in particular to governmental institutions, local communities, and other citizens at risk of flooding?

This paper begins by presenting the analytical frameworks and methods in Sections 2 and 3: the risk management cycle and a screening of the BUI landscape for answering the first, and the proximity concept and an in‐depth analysis of three study sites for answering the second research question. The results in Section 4 first map the current scope of flood BUIs in Europe, North America, and Australia and then spotlight the three study sites, assessing how BUIs tie in with other activities and actors. Therein, we illustrate the spectrum of self‐organised to formalised BUIs, that is, BUIs independent from or supervised by governmental authorities. Section 5 provides a conclusion on future roles of BUIs in regards to existing institutions.

## 2 ANALYTICAL FRAMEWORKS

We employ two analytical frameworks to describe and classify BUIs: first, we map BUIs' activities to the *prevention, protection, preparedness, response, and recovery stages of the classic risk management cycle* (EU, 2004; see Table 2). BUI activities in lobbying to governmental bodies are mapped to the stages specific for the contested policy; for example, a BUI that negotiates the designation of flood risk zones with the local authorities is mapped to the prevention stage. Using the risk management cycle framework, we illustrate typical activities of BUIs, and how these activities complement existing institutions in flood risk management.

As second analytical framework, the concept of *proximity* allows us to better understand how stakeholders operate in a collaborative setting such as flood risk management (Boschma, 2005; Huber, 2012; Lundquist & Trippl, 2013). Proximity encompasses geographical distance as well as involvement in a shared network (Torre & Rallet, 2005). The proximity lens enables analysis of the partnership arrangements that BUIs establish with local stakeholders. Here, we differentiate between the institutional, relational, and social dimension of proximity, which are common distinctions in proximity theory, as they describe the stakeholder interrelations emphasised in risk governance (Thaler, Priest, & Fuchs, 2016). Table 1 lists indicators of how each proximity dimension can be evaluated in a particular BUI (higher levels of proximity imply that a BUI is more likely to achieve its objectives).

**Table 1:** Analytical criteria for the proximity framework

Proximity dimension	Indicators
Institutional proximity (between the BUI and the public administration)	The BUI's agenda and arguments enter and shape formal decision processes and lead to consensus building instead of unilateral decision‐making
	The BUI receives support from authorities (e.g., funding, resources, sharing of knowledge)
	The BUI's area of influence overlaps with the boundaries set out by the rule of law or political practice
	Roles and responsibilities of the BUI are defined in relation to the roles of other formal actors
Relational proximity (between the BUI and its local community or adjacent regions)	The BUI has access to material and non‐material support from local networks
	The BUI shares coherent aims with other actors
	The BUI coordinates with and complements parallel community processes
	The BUI exchanges developments, objectives and concepts with other actors (social learning)
Social proximity (among BUI members)	BUI members share coherent aims
	BUI members trust each other
	Low variation and high interaction between BUI members
	BUI includes everyone who is affected and wishes to participate
	Key personnel hold defined roles and act in a transparent and accountable way

*Institutional proximity* refers to the relationship between a BUI and governmental institutions in flood risk management. Institutional proximity describes the political influence a BUI exerts, and to what extent the issues advocated by a BUI are taken up by those formally in power. Institutions set the “rules of the game” within society; these rules govern individual behaviour and structure social interactions towards joint decisions (North, 1990; Raschky, 2008). A BUI that performs well within the institutional ruleset may achieve a position in the risk debate that goes beyond petitioning governmental decision makers (Albrechts, 2013).

*Relational proximity* describes how deeply a BUI is embedded in its home community as well as its relations to adjacent regions, such as those located in the same river catchment. Relational proximity also reflects social capital as well as language, experiences and worldviews shared with other citizens (Bourdieu, 1986; Coleman, 1990); these informal threads of the social fabric may give a BUI additional leverage in the risk debate.

*Social proximity* relates to the size and quality of the network among BUI members. Citizens who engage in a BUI form interpersonal linkages based on friendship and trust (Huber, 2012). The lifetime of a BUI critically depends on the personal relationships between its members (Balland, 2012). Local leaders or social entrepreneurs, such as flood wardens or chairmen, are regarded as crucial to build up and maintain expertise and commitment in a BUI (Mintrom & Vergari, 1996).

In both analytical frameworks, stages or dimensions may overlap to some degree. For example, if a BUI sets up a depot for emergency materials (protection stage) and conducts training in how to use them (preparedness stage), BUI members will probably also apply these procedures together when a flood strikes (response stage). Supra‐regional administrative bodies may affect both institutional and relational proximity; or social and relational proximity may overlap, if BUI members hold multiple community roles.

## 3 METHODS

**Figure 1:**
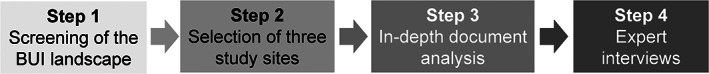
Four‐step approach

We apply a four‐step approach to investigating BUIs in flood risk management (Figure 1): first, an extensive screening based on scientific literature, online sources (including media reports and social media platforms) and expert networks was conducted to identify the current diversity of BUIs. This screening was compiled with the objective of achieving maximum variation within the sample, a common approach in qualitative research (Anderson, 2010). The initial online search used predetermined keywords to capture the citizen‐driven nature of initiatives, including “bottom‐up flood mitigation,” “flood action group,” and “Bürgerinitiative Hochwasserschutz.” Subsequently, search terms were added, refined and translated to improve coverage. A content analysis structured the characteristics and activities of each identified BUI. Because of language barriers with the authors, the screening was limited to information in English and German. The screening adopted a scoping approach (Arksey & O'Malley, 2005), which does not amount to a systematic review. This was due to difficulties in developing universally valid criteria for BUIs and in retrieving an exhaustive set of BUIs, which are often short‐lived, sparsely documented and appear only sporadically in public communication channels. The screening sample, however, is neither comprehensive nor representative and thus may not reflect the actual distribution of BUIs and their activities. Therefore, we may not report any numerical estimates on the prevalence of BUI characteristics or activities. Nevertheless, the screening provides valuable insights on the general activities and orientation of the BUI field.

In the second step, three study sites were selected from the screening to assess the activities of each BUI in depth as well as its relationship with governmental authorities, its local community, and among its members. As a third step, a document analysis for each study site compiled legal documents, flood risk management plans, information available on BUI websites, and media reports. In the final step, semi‐structured qualitative interviews with three to five experts per study site validated and contextualised the findings, and captured aspects not covered by written sources. Interview participants were recruited from among local key actors in administration, academia and the BUIs themselves (see Table A1 in Appendix).

## 4 RESULTS

### 4.1 Landscape of flood BUIs

Our screening resulted in 70 BUIs across eight countries, among which we found BUIs to be most prevalent in Germany and the United Kingdom (Table A2 in Appendix; the italicised terms and IDs given below refer to BUIs and headings in this table). Although the BUI landscape appears to be highly fragmented, any particular BUI can be characterised by whether or not it is a pressure group, its triggers for formation, its activities within the risk management cycle, and its geographical scope.

#### 4.1.1 Pressure groups

The key objective of BUI pressure groups is to oppose plans or actions taken by governmental authorities. The existence of pressure groups may point to a failure in flood risk governance, particularly if citizens feel excluded from the policy debate or if they think that their fundamentally different, possibly even incompatible, views do not receive due consideration. Usually, BUI pressure groups seek media attention, publish much of their information online and mobilise local residents for protest activities. One archetypical example is the *Initiative Hochwasserschutz Eferdinger Becken* (ID4), which publishes counterstatements online, and organises protest marches or joint opposition at risk information events held by the authorities. Overall, our screening indicates that pressure groups seem to be more widespread in Germany and Austria, but less common in the United Kingdom and the United States. This might be due to more integrative governance processes in the latter countries, or a less prevalent attitude of entitlement to public flood protection.

#### 4.1.2 Triggers of formation

Flood BUIs typically emerge out of frustration with current flood risk policies, the occurrence of a specific flood event, or an opportunity offered by civic engagement projects or availability of funding. *Frustration* often arises from an unsatisfactory status quo, from a perceived lack of urgency in the authorities, or from planned public protection schemes that substantially impact the livelihoods of residents. The *Bürgerinitiative Hochwasser Würschnitztal* (ID24), for instance, has been frustrated by the slow progress of the planning and approval of local flood protection. Other frustration‐related triggers are nontransparent planning processes (e.g., *Interessensgemeinschaft Inntal*, ID3) or ongoing conflicts about the location of planned flood defence systems (*Bürgerinitiative Landschaftsverträglicher Hochwasserschutz Hexental*, ID18). Specific, often catastrophic, *flood events* are another common trigger. The *Sedgeberrow Flood Group* (ID43), for example, was founded in the aftermath of the 2007 flood event, when existing management strategies proved insufficient. Other BUIs seized *opportunities* offered by local authorities, such as the *Purley Flood Defence Group* (ID48), which was set up after the council contacted interested individuals. Opportunities can also arise through governmental funding, as it happened with the *Bodenham Flood Protection Group* (ID55), which received money from the Parish Council in its first year to buy flood defence tools and later transitioned into a self‐sustaining group.

#### 4.1.3 Activities within the risk management cycle

The activities of BUIs vary widely across the five stages of the risk management cycle. With respect to the prevention stage, BUIs push for revisions of hazard maps (e.g., *Interessensgemeinschaft Inntal*, ID3), oppose further surface sealing caused by real estate development (e.g., *BürgerInnen‐Initiative Hochwasserschutz Laakirchen*, ID8), identify potential retention areas (e.g., *Initiative Hochwasserschutz Buldern*, ID21) or carry out petitions against building on flood plains (e.g., *Glastonbury Northload Bridge Residents Flood Risk Action Group*, ID59). BUIs also engage intensively in the protection stage. The *Brompton Flood Prevention Group* (ID57) for instance, has implemented a series of natural defences against flash floods, paid for by villagers. The *Hands on Nashville: Waterway Cleanup, Restoration and Emergency Response* initiative (ID68) helped to remove debris from local streams and creeks. The *Tadcaster Flood Action Group* (ID51) is particularly active in the preparedness stage; it has invested in an early warning and monitoring system, and trains volunteers in the use of equipment like radios and flood pumps. Other BUI preparedness activities include raising awareness of what residents should do before, during and after a flood event (e.g., *Bürgerinitiative Andritz*, ID6), developing community flood plans (e.g., *Purley Flood Defence Group*, ID48) and setting up flood stores (e.g., *Todmorden Flood Group*, ID50). Typically, flood stores are situated in key areas and provide emergency equipment such as shovels, axes, flashlights, walkie‐talkies, first aid kits, tents, or generators. A common BUI activity spanning across the preparedness and response stages is the introduction of flood wardens (e.g., *Mytholmroyd Flood Group*, ID46). Flood wardens are community volunteers who are trained to watch a section of a river and report critical conditions to early warning systems. They also educate locals about flood risks and establish lines of communication to emergency and rescue services. BUI activities related to the response phase include setting up databases of people who would need or could provide help (e.g., *West Virginia Flood Relief Community Connection*, ID64), sandbagging and supporting evacuation of residents (*Tadcaster Flood Action Group*, ID51), and providing food and cleaning materials (*arche noVa Fluthilfe*, ID12). Other flood BUIs focus on the recovery stage: the *Lancashire Flood Recovery Fund* (ID40) distributes charitable donations and grants to flood victims. The *Community Emergency Response Team* (ID42) helps with cleaning out affected homes, supplies cleaning products and replacement furniture, and offers moral support. Many BUIs engage in several stages of the risk management cycle. The *Todmorden Flood Group* (ID50), for instance, promotes risk awareness among households, maintains flood stores, assists with insurance queries and stays in touch with relevant agencies to develop and implement local flood plans.

#### 4.1.4 Geographical scope

Most BUIs address issues at the local level, where the consequences of flood hazards are mainly experienced. However, it seems that BUIs increasingly realise that cross‐regional collaboration allows them to tackle flood hazards more effectively and to create additional political leverage. The *Zusammenschluss der Interessengemeinschaften—Kontra Polder an der Oberen Donau* (ID19) is such an example of regional cooperation seeking to align interests along the Danube River. Expanding beyond catchment‐wide cooperation, the *National Flood Forum* (ID60) is dedicated both to establishing a country‐wide network of community groups who share information and experience, and to channelling political influence. We only identified one single BUI that extends its activities beyond national borders: *arche noVa Fluthilfe* (ID12) supports communities in Germany and the Czech Republic in reducing future flood risks and recovering from catastrophic floods.

Our screening also reveals specific interrelations between BUI characteristics. Frustration, in particular, appears to fuel the formation of pressure groups. Pressure groups usually only address prevention and protection stages and limit their geographical scope to local issues. Numerous BUIs that are active in the recovery stage seem to have been triggered by previous flood events, probably because the recovery issue is more salient in communities with direct flood experience. BUIs concerned with protective measures often extend their geographical scope to the regional level, presumably because they recognise that successful risk reduction also depends on actions taken in up‐ and downstream communities. The screening further shows that BUIs from Germany and Austria mainly focus on the prevention and protection stage and try to shape flood mitigation plans by the government. Most British and American BUIs, on the other hand, follow a hands‐on approach, trying to improve community resilience by means of low‐cost, improvised and easy‐to‐deploy measures.

### 4.2 Study sites context

Based on the screening, we zoom in on three BUI study sites to illustrate the full scope of missions and origins of a BUI: the C*ockermouth Flood Action Group*, born out of the need for self‐reliance, as local risk management institutions are absent; the *Bürgerinitiative Hochwasserschutz Übigauer Insel*, established to co‐design flood protection at eye‐level with the authorities; and the *Flutschutzgemeinschaften Hamburg*, obligated by law to fulfil former state roles in flood preparedness and response at the building level. The study sites were purposefully selected as contrasting cases to span BUIs that are (1) active in various stages of the risk management cycle; (2) recently established or grown over a long time; and (3) self‐organised grassroots movements triggered by civic frustration or formalised groups initiated by the government.

#### 4.2.1 Cockermouth flood action group (United Kingdom)

The civil parish of Cockermouth is located in the highly frequented Lake District tourism area. The two rivers Cocker and Derwent merge close to the town centre. Cockermouth has been flooded three times in rapid succession—in 2005, 2008, and 2009. The 2009 event affected the whole community and flooded more than 693 residential houses and 225 businesses (Cumbria Resilience, 2011).

The first community engagement emerged as a small self‐help group of local residents after the 2005 flood. The initiative did not spread to the whole town, though. The 2008 flood event re‐stimulated citizen action to resolve surface runoff in the Gote Road. Nevertheless, the *Cockermouth Flood Action Group* was only created after the devastating 2009 flood event. Local stakeholders began to exercise considerable pressure in the local flood policy discussion. The community worried about excessive insurance premiums for private households or businesses, or being denied a bank loan if no longer able to secure insurance. In addition, citizens perceived a lack of support and interest from political representatives after the flood event. Consequently, frustration and mistrust towards the civil authorities built up. The formation of the Cockermouth Flood Action Group was assisted by the British *National Flood Forum*, a UK‐wide BUI, established in 2002, that supports local BUIs, both in disaster recovery and implementing local preventive measures. Furthermore, the National Flood Forum lobbies local and national governmental bodies, giving people at risk a stronger voice in policy decisions (Thaler & Levin‐Keitel, 2016).

#### 4.2.2 Bürgerinitiative Hochwasserschutz Übigauer Insel (Germany)

Übigau is an urban district of Dresden located directly at the River Elbe. The *Bürgerinitiative Hochwasserschutz Übigauer Insel* was launched one year after Übigau was heavily hit during the Elbe flood in June 2013. After this flood, residents recognised the need to prepare for future floods, especially regarding first responder reaction times, as many resources were wasted and sandbags inefficiently deployed because of coordination problems.

The BUI aims to enhance cooperation with responsible government agencies, which will point out potential improvements to existing dams and emergency plans. The action group is funded by private sponsors and receives no public money. Its activities primarily target the district of Übigau; all citizens with personal ties to this district can join the effort.

The *arche noVa* association has contributed external expertise to the development of emergency plans for Übigau. Arche noVa—Initiative for People in Distress—is an international aid organisation specialising in water, sanitation, and hygiene education. Dresden's city government actively seeks non‐bureaucratic collaboration with residents on future flood protection. This cooperative effort started in a joint risk analysis and awareness‐raising session in 2016 with all emergency services, the action group and representatives from the local administration.

#### 4.2.3 Flutschutzgemeinschaften Hamburg (Germany)

The urban area HafenCity in Hamburg faces severe risks from tidal floods when storm surges from the North Sea travel up the estuary of the River Elbe. Protecting this area with conventional floodwalls would have incurred substantial costs and delays in real estate development. Instead, and complementary to strict building codes, the City of Hamburg passed a regulation in 2002 requiring building owners in this area to put into effect a partnership for flood protection for each residential building (called *Flutschutzgemeinschaft*, FSG; LSBG, 2012; Senat Hamburg, 2002). By law, FSGs have to document, maintain, exercise and execute flood protection at their respective buildings. The members of an FSG nominate a *Flutschutzbeauftragter* out of their circle as speaker and overseer. This person's position is unsalaried; however, the actual workload is rather small (Fellmer, 2014a; Mees, 2013). The majority of FSGs subcontract their duties to security agencies or property managers, though.

The introduction of FSGs by law is in line with the strongly regulated and hierarchical governance approach in Hamburg (Fellmer, 2014b; Mees, 2013). Residents take part in decision making only indirectly, through elected representatives; still, according to Mees, Driessen, and Runhaar (2014), they are satisfied with the division of responsibility and the level of safety. The introduction of FSGs amounts to shifting duties formerly carried out by the government to the affected citizens. An FSG is subsidiary to the flood management authorities and subject to their inspection. FSGs have to take self‐reliant action when a flood strikes and cannot rely on any governmental backup apart from emergency rescue services.

### 4.3 Activities of the study sites within the risk management cycle

The C*ockermouth Flood Action Group* influenced the decision process in the design of the local flood alleviation scheme (protection stage; Table 2). Currently, the BUI is playing a central role in the formation of the Cockermouth Emergency Response Group; therein, local citizens are involved and supported in the preparedness, response and recovery stages. However, the BUI is headed by a small circle of individuals and hardly reaches out to engage the larger community. The BUI has had to acquire technical knowledge from external consultants, an option which is only open to initiatives that command adequate financial resources. The BUI encouraged the Environment Agency, a national authority, to revise its original planning for flood protection despite higher costs. The revised scheme caters to the needs and interests of the town, providing economic benefits, and to individual households by means of a self‐raising barrier which offers the aesthetic benefit of an unimpaired view of the river. The BUI is also active in lobbying at the national level. However, these lobbying activities focus on promoting local Cockermouth interests, which implies a lack of solidarity and potential conflicts with neighbouring communities.

**Table 2:** Assignment of study site activities to the stages of the risk management cycle

Stage	Cockermouth	Übigauer Insel	Hamburg
Prevention	None	None	None
Protection	Developing a local flood risk management plan (in cooperation with the national government)	Identification of potential improvements to existing protective measures	Documentation of structural facilities in the building
			Yearly inspection of flood doors and evacuation routes
Preparedness	Encouraging residents, to adapt their households, through face‐to‐face communication, organising round tables and discussions forums, leaflets, web‐site etc.	Development of an emergency plan	Setting up and updating an emergency plan detailing technical operations, communication and evacuation
		Risk analysis and risk awareness workshops with the emergency services, for example, site visits, communication training	Yearly flood emergency training with residents
			Building risk awareness among residents, for example, with flood bulletins in the hallway
Response	Improved flood alert within the community	Reducing the reaction time of first responders	Timely alert during a flood event
	Support of evacuation centres		Closing flood doors and activating pumps
Recovery	Support in filling insurance claims	None	None

The principal activities of the *Bürgerinitiative Hochwasserschutz Übigauer Insel* focus on the protection and preparedness stages—namely, improving communication with the relevant governmental departments for flood protection. Under the guidance of arche noVa and the City of Dresden, the BUI has co‐produced an emergency plan which includes a detailed documentation of staff and materials that can be mobilised in a flood crisis. During the response to the 2002 and 2013 floods, poor cooperation with the authorities became apparent. To avoid similar problems in the future, the BUI has established contact persons for direct communication with city officials. These contact persons serve as intermediaries during a flood emergency, but also promote risk awareness among residents. Furthermore, the BUI works on branching out communication with other action groups in Dresden.

While nominally an independent group, in practice the *Bürgerinitiative Hochwasserschutz Übigauer Insel* undertakes its activities as an auxiliary under the leadership of the governmental authorities. In order to maintain the BUI's operational capacities as a full‐fledged emergency unit, especially over long periods without flood events, city officials train BUI members and provide specific guidance on the implementation of protective measures. During an actual flood response, the BUI is put under governmental command.

The main activities of the *Flutschutzgemeinschaften Hamburg* (FSG) revolve around maintaining and operating flood doors that seal off the ground floors of buildings (protection and preparedness stages). In addition, FSGs ensure that residents will comply with evacuation procedures (response stage). Each FSG has to submit yearly reports to the authorities; the authorities review these reports and, if deemed necessary, verify the practical implementation in site visits. This assignment of the operational role to the FSG and of the oversight role to the authorities is fully accepted among all involved actors. FSG duties can be considered a well‐defined, integral part of the overall flood management scheme in the HafenCity area.

However, in two respects, the actual role of FSGs falls short of the expectations put forward in the underlying legislation. First, the law which originally installed the FSG concept intends those groups to also raise flood awareness among residents as a side benefit (Mees, 2013). In those cases where residents disengage from their FSG role by subcontracting it to external companies, this side benefit is presumably diluted due to diminished citizen involvement. However, in the current view of the authorities, it would be unrealistic to expect this side benefit in an anonymous urban setting that hardly lends itself to promoting risk awareness among a highly fluctuating populace. Second, although the obligations of the operative personnel in FSGs are clearly set out, they are not empowered with the necessary know‐how to fulfil these obligations. The FSGs have to acquire their practical knowledge from learning‐by‐doing, from occasional instructions by flood door manufacturers, or by approaching pertinent experts. The flood management authorities criticise the wide variation in FSG qualifications but do not yet offer a dedicated training program.

### 4.4 Proximity of the study sites to other stakeholders

Institutional proximity reflects the relationship between a BUI and flood management authorities. The *Cockermouth Flood Action Group* features the highest institutional proximity of the three study sites. The turning point in the BUI's relationship with the Environment Agency as responsible authority occurred when the BUI changed its own self‐concept from a pressure group to an organisation with professional competences and with the aim of collaborating with the national authorities. The BUI even subcontracted the first flood risk management plan for the town to an external consultant company at their own expense. For its part, the Environment Agency shifted from solely carrying out flood engineering towards a project management role, focusing on negotiating and networking. This change of roles endowed the Cockermouth BUI with legitimacy and respectability, and consequently opened up a conversation about the design of flood alleviation Schemes. A similar agreement is underway in the case of the *Bürgerinitiative Hochwasserschutz Übigauer Insel*. Here, the authorities are aware and appreciative of the BUI, reflecting the level of trust in the group and its political voice. However, the BUI's actions do not manifest as co‐production among equals, but are subordinated governmental leadership and the expert support of arche noVa. Contrastingly, the *Flutschutzgemeinschaften* case amounts to a highly formalised civic duty imposed by law. Civil servants and the FSGs communicate only through standardised processes such as updating address information, filing reports, and inspecting selected buildings. Now, though, more than a decade after the original FSG regulation was passed, the authorities have developed informal procedures for quality assurance which go beyond a narrow supervisory role. City officials are sensitive towards minor lapses in FSG activities (such as poor technical competence or delayed notification if a flood door was closed); if they detect signs of negligence, they seek a clarifying discussion.

Relational proximity refers to how a BUI interacts with other stakeholders within the local community. In the *Cockermouth* case, the BUI channels community needs and facilitates local decisions that reflect these needs. In the flood defence scheme along the Rubby Banks Road, residents were adamant about retaining an open view of the river. The BUI organised local engagement and subsequently encouraged the Environment Agency to adapt their plans. The strong feeling of solidarity after severe flood events, already present in the *Übigau* district, has been intensified by the BUI's establishment. Local businesses in the district also became involved and promised assistance during future events. The Übigau BUI's community relations are closely connected to the role of arche noVa, who used Übigau as a showcase for a new handbook for facilitating community processes. Besides supporting the development of an emergency plan, arche noVa provided negotiating power and advice in the debate with civil servants and emergency service commanders. While the specific geographical layout of the Übigau district prohibits direct cooperation with neighbouring regions during catastrophic events, cross‐district collaboration is encouraged through an exchange of information. *Flutschutzgemeinschaften*, on the other hand, are largely disconnected from their local communities. Knowledge sharing between FSGs only occurs if one and the same person or company leads the FSGs of several buildings. FSGs have no discernible ties to other community initiatives; however, to some extent this can be attributed to the anonymous setting of a recently‐founded urban quarter, where close neighbourly networks and risk awareness, which could sustain an FSG, have not (yet) emerged.

Social proximity describes the relationships between BUI members themselves. Interactive arrangements within the *Cockermouth* BUI ensure sufficient access to information and decision‐making processes for all members. The BUI has recruited members with different backgrounds and knowledge, partly resulting from its origin as a self‐help group after previous flood events, and partly from continuous community interaction over the years. However, the BUI depends heavily on its organisation committee of local leaders, who manage all interactions with the local community and national authorities. The leadership of the *Übigauer Insel* BUI also consists of a core team. This requires considerable effort in reporting periodically to all concerned residents; however, in line with the BUI's objectives, this organisational structure ensures that external actors have direct access to a contact person at any time, even in times of crisis. The social proximity of the *Flutschutzgemeinschaften* is less pronounced and further weakened by the tendency to subcontract FSG duties to external companies. This contractual circumventing of actual citizen engagement may have been predisposed (or at least facilitated) by the original law that made FSGs mandatory: describing the FSG duties exactly but leaving open who may execute them made this function transferable to third parties. Thus, many FSGs are not maintained by interpersonal trust among their members, but by monetary transactions for the provision of flood protection services. Conducting FSG duties is currently evolving into a dedicated business model for building management services in Hamburg. If this tendency continues, FSGs might fully lose their BUI character and turn into a vehicle for the privatisation of flood risks.

## 5 DISCUSSION AND CONCLUSIONS

BUIs are rapidly evolving in the flood policy arena, and the present study can only provide a snapshot of their current scope in Europe, North America and Australia. The recent emergence of flood BUIs coincides with a general trend of citizen initiatives challenging state governmentality, such as urban greening initiatives or community photovoltaic power plants (Cloutier, Papin, & Bizier, 2018; Hatzl, Seebauer, Fleiß, & Posch, 2016). BUIs offer a promising avenue to putting into practice the risk governance objective of co‐producing natural hazard management with affected citizens (Nye, Tapsell, & Twigger‐Ross, 2011; Scolobig & Pelling, 2016). As bridging organisations, BUIs may bundle individual views into a joint agenda, thereby allowing the citizenry to enter partnerships with state institutions. Ideally, BUIs channel workforces and expertise into localised, effective and accepted plans to manage current and future flood risks (Thaler & Levin‐Keitel, 2016).

BUIs are active throughout all stages of the risk management cycle, often taking over roles that the authorities are not (or no longer) willing or able to fulfil (see Table 2). The wide range of BUI activities, as outlined in the screening, may be a stimulus to flood risk managers who seek innovative ways to involve citizens. However, as is evident in the Cockermouth and Übigauer Insel study sites, for BUIs to effectively enter existing management structures, both BUI members and relevant authorities need to rethink their traditional roles: while citizens need to leave behind protest motivations and build specialist expertise, authorities have to abandon paternalistic mindsets and widen their policy toolbox from technical fixes to social and organisational innovations. If both sides revise their traditional positions, reciprocal respect and shared language may set the stage for mutually beneficial collaboration (Nye et al., 2011).

Our results show that BUI constellations cover a spectrum, from self‐organised grassroots movements (e.g.,the Cockermouth study site), to initiatives that grow out of formal citizen hearings (e.g., opportunity as a trigger for formation, see Section 4.1.2), to legal arrangements where the authorities outsource their former protective tasks to the residents at risk (e.g., the Hamburg site). Self‐organised movements may benefit from their core circle of committed members with high relational and social proximity. By merit of their cohesion as a group and embedding in their community, they can keep up their efforts over a long time and adapt to changing political, social or hazard environments. However, some friction can be expected until the movement clarifies its role in the policy arena. In contrast, the strength of BUIs enacted by law lies in their institutional proximity: the legal grounds clearly define their role in relation to established institutions. On the other hand, as soon as the legal obligation or the monetary incentive for subcontractors is discontinued, those BUIs might swiftly break down, since they cannot draw on interpersonal trust as a foundation for their continued existence (see Table 3). Still, the *Flutschutzgemeinschaften* example underlines that even legally forcing citizens to band together can relieve responsibilities of the state, although it does not follow the higher (and sometimes romanticised) call for participatory, non‐hierarchical risk governance.

**Table 3:** Proximity assessment of study sites

Dimension of proximity	Cockermouth	Übigauer Insel	Hamburg
Institutional proximity	Strong engagement and impact in decision‐making, based on incorporation of internal and external knowledge, such as recruitment of specialists, collaboration with private consultant groups, or informal contacts with the national government	Support from authorities was intensified after the last event	Responsibilities are clearly defined by law
	The BUI's role shifted from a lobby group to a professional dialogue partner	Routines are established for working together with local emergency services in the case of a flood event	Top‐down relationship: Authorities act as supervisors, FSGs act as operational subsidiaries
	Environment agency remains a key player as supervising authority	Strong engagement from authorities for a long‐term cooperation with the BUI	Authorities do not offer dedicated support to FSGs, aside from water level warnings and flood information leaflets available to all residents
Relational proximity	Local actors contributed more than £ 1.1 million to the scheme	The BUI receives construction machinery and sand for filling sandbags from local networks	FSGs are not active beyond their legal mandate
	Temporary increase of the local council tax showed strong approval from the community	The BUI's influence is focused on its city district	No FSG umbrella organisation exists
	The BUI was strongly supported by the National Flood Forum, in terms of transfer of knowledge and technical expertise	No involvement in other community processes	Build‐up of knowledge and operational synergies only if the same subcontractor works for several buildings or FSGs
		Future joint activities with action groups in neighbouring city districts are planned	If the FSG consists of a sole building owner, then he may draw on an extended network of property management
		Core support role by arche noVa	
Social proximity	Low variation among the BUI speakers	The BUI speakers also act as opinion leaders within the group	Members have a contractual, not a personal relationship
	Personal relationship to the BUI	High solidarity and mutual help among members in the case of a flood event	High variation among residents hinders the formation of a social network underpinning the FSG
	High degree of common goals and vision within the members of the BUI, as well as within the community	Everybody is invited to join the action group	

The diversity of activities and constellations observed in our screening speaks to the flexibility of BUIs as an additional instrument of flood risk governance. BUIs seem remarkably agile in terms of their duration and scope, particularly if they are self‐organised groups active in the preparedness, response and recovery stages: they can be established quickly, maintained for a limited period, and disbanded as soon as their mission is accomplished. They can easily reorient their agenda if local needs shift or previously silent population segments raise their voices. They are unconstrained by the legal and administrative rulesets with which governmental authorities have to comply. The Cockermouth BUI demonstrates this flexibility, as it iteratively changed its mission three times after experiencing a flood event. Therefore, state actors may consider BUIs a highly adaptable, albeit at times unpredictable, tool for change that may cover jurisdictional blind spots or underfinanced areas in all stages of the risk management cycle.

Extending its reach beyond the home community seems important for realising the interests of a BUI. The screening shows that many BUIs have a narrow, local geographical scope. Those BUIs may find it difficult to compete against institutional players drawing on wider expertise and circles of influence. Most BUIs lack external support in terms of technical knowledge transfer or legal backing. Presumably, none of the three study sites would have established itself successfully in the risk policy arena, had it not received a formalised legal status (regulation by the City of Hamburg) or been linked to national NGOs (Cockermouth with National Flood Forum, Übigauer Insel with arche noVa). Therefore, BUIs can be advised to seek collaboration with other civic actors in their catchment area, in order to have a stronger public voice and to obtain additional resources and ideas. National governments wishing to promote the development of BUIs should avoid scattering seed financing over small BUIs. Instead, supporting umbrella organisations may yield leverage and access to the agenda setting of numerous BUIs nationwide. However, umbrella organisations should navigate their role carefully: If they focus to heavily on political lobbying, they may lack the grassroots appeal for emergent pressure groups. If they provide too detailed practical expertise, they may undermine the self‐reliance of local BUIs, as citizens overwhelmed by the legal and engineering complexity of the flood topic may quickly hand over responsibility to the external experts (e.g., as indicated in the relation between the Übigauer Insel BUI and arche noVa).

Regardless of their value in bringing collaborative risk governance into practice, BUIs may create parallel political structures that are not democratically legitimised (Allmendinger & Haughton, 2009; Cloutier et al., 2018). A few outspoken community members might become BUI chairmen; local elite groups, especially, are likely to have the skills necessary to take a position of leadership (Begg, 2018; see Table 3). Civil servants might be tempted to build a personal relationship with a BUI contact without being aware whether this person actually represents the silent majority of all concerned citizens. If the BUI as a non‐state actor dominates the risk debate, it may undermine the standing of state actors that are directed, controlled and held responsible by elected representatives. However, in our three study sites we did not find any indications that BUI spokespeople had overstepped their mandate. The Übigauer Insel BUI even considers inclusiveness a central objective of the initiative. Nevertheless, all risk governance actors should be sensitive to the question of whether BUIs and their representatives have the full backing of the communities they claim or intend to represent.

Another caveat arises from the descriptive character of this study. At present, the BUI landscape seems too diverse and too fragmented to allow for more than mapping the overall directions taken; however, as soon as BUIs have consolidated their roles in the risk governance arena, we would welcome future studies to conduct a comprehensive review and provide a quantitative assessment on the prevalence of certain BUI characteristics and activities. Until then, state funding for citizen participation could be directed towards quasi‐experimental field studies comparing which BUI format works best in which local context.

Finally, the capability of BUIs to tackle environmental problems should not be overestimated. BUIs are not necessarily more efficient than centralised institutions if they merely act as lobbying organisations to influence decision makers instead of contributing new ideas; if they promote only their local agenda without partnership over communal borders; or if they predominantly advance the interests of the more affluent and fail to support deprived communities. It remains a key challenge for risk governance to weigh, in each particular case, whether transferring responsibilities to local communities or enacting top‐down procedures results in a better ratio between costs, inclusiveness, accountability and effective risk reduction.

## Appendix

**Table A1:** Organisations of interviewed experts

Study site	Organisations of interviewed experts
Cockermouth	National Flood Forum Cockermouth Flood Action Group Environment Agency Department for Environment, Food & Rural Affairs Cumbria County Council
Übigauer Insel	Bürgerinitiative Hochwasserschutz Übigauer Insel Arche noVa – Initiative for People in Distress City of Dresden, Environment Services
Hamburg	Hamburg State Company for Roads, Bridges and Waterways, Department of Dike Defence and Monitoring City of Hamburg, Internal Services, Disaster Control and Civil Protection HafenCity University Hamburg, Resource Efficiency in Architecture and Planning Research Group *Flutschutzbeauftragte* of selected *Flutschutzgemeinschaften* (provided by courtesy of Mareike Fellmer)

**Table A2:** Screening of bottom‐up initiatives in flood risk management

			Triggers of formation			Activities within the risk management cycle					Geographical scope				
Bottom‐up initiative (BUI)	Country	Pressure group	Frust‐ration	Flood event	Oppor‐tunity	Pre‐vention	Pro‐tection	Pre‐paredness	Response	Recovery	Local	Regional	National	Inter‐national	Reference ID
Ulupna to Barmah Floodwatch	AUS	No		x				x				x			1
Numurkah flood action group	AUS	No		x				x			x				2
Interessensgemeinschaft Inntal	AUT	Yes	x			x	x					x			3
Initiative Hochwasserschutz Eferdinger Becken (OÖ)	AUT	Yes	x				x					x			4
Initiative für ökologischen und nachhaltigen Hochwasserschutz ‐ Aist	AUT	Yes	x				x					x			5
Bürgerinitiative Andritz	AUT	Yes	x				x	x			x				6
DV‐Donau Verein für nachhaltigen Natur‐ und Hochwasserschutz an Donau und Zubringern e.V.	AUT	No			x	x	x						x		7
BürgerInnen‐Initiative Hochwasserschutz Laakirchen	AUT	Yes	x			x	x				x				8
Team Österreich	AUT	No			x				x	x			x		9
Team Weissenkirchen	AUT	No			x				x		x				10
Interessengemeinschaft Reuss für einen vernünftigen Hochwasserschutz	CHE	Yes	x				x				x				11
Arche noVa Fluthilfe	DEU	No			x			x	x	x				x	12
Hochwasserschutz Elbe‐Mulde	DEU	No		x				x				x			13
Hochwassernotgemeinschaft Rhein	DEU	No		x		x	x	x				x			14
Hochwasserschutz‐Initiative Elbe‐Havel‐Winkel	DEU	No		x		x	x	x				x			15
Bürgerinitiative Naturnaher Hochwasserschutz Selke	DEU	Yes	x			x	x					x			16
HWS ‐ Hochwasserschutz‐Initiative am Niederrhein	DEU	Yes	x				x	x				x			17
Bürgerinitiative landschaftsverträglicher Hochwasserschutz Hexental	DEU	No	x				x					x			18
Zusammenschluss der Interessengemeinschaften ‐ Kontra Polder an der Oberen Donau	DEU	Yes	x				x					x			19
**Bürgerinitiative Hochwasserschutz Übigauer Insel**	DEU	No	x	x			x	x	x		x				20
Initiative Hochwasserschutz Buldern	DEU	No		x		x	x				x				21
Bürgerinitiative Hochwasser Aken (Elbe)	DEU	No	x				x				x				22
Bürgerinitiative für eine verträgliche Retention Breisach/Burkheim	DEU	Yes	x				x				x				23
Bürgerinitiative Hochwasser Würschnitztal	DEU	Yes	x				x				x				24
Bürgerinitiative für Hochwasserschutz Kobern‐Gondorf	DEU	Yes	x				x	x			x				25
Bürgerinitiative Hochwasser Nünchritz 2013	DEU	Yes	x			x	x				x				26
Bürgeriniiative der Ortsteile im Osten von Erfurt	DEU	No	x	x		x	x				x				27
Bürgerinitiative Hochwasserschutz für die Isarsiedlung	DEU	Yes		x			x				x				28
Buergerinitiative Hochwasser‐ und Naturschutz Altrip	DEU	Yes	x				x				x				29
Bürgerinitiative Rückhaltebecken Dietingen	DEU	Yes	x				x				x				30
Interessengemeinschaft Hochwasserschutz Ja‐ Polder nein	DEU	Yes	x				x				x				31
Bürgerinitiative Rettet das Donauried	DEU	Yes	x				x				x				32
**Flutschutzgemeinschaften Hamburg**	DEU	No			x		x	x	x		x				33
Bürgerinitiative Grimma	DEU	Yes		x	x		x				x				34
Bürgerinitiative Hochwasser ‐ Altgemeinde Rodenkirchen	DEU	No		x		x	x	x			x				35
Ungebundene Helfer während der Flut Dresden 2013	DEU	No		x					x		x				36
Freuchie flood action group	GBR	No		x			x	x			x				37
Isbourne catchment group	GBR	No			x	x	x					x			38
Flooding on the levels action group	GBR	Yes	x	x			x					x			39
Lancashire flood recovery fund	GBR	No		x					x	x		x			40
Pang Valley flood forum	GBR	No			x		x	x				x			41
Community emergency response team	GBR	No		x						x		x			42
Sedgeberrow flood group	GBR	No		x		x	x	x	x		x				43
Cockermouth emergency response group	GBR	No		x				x	x		x				44
Stonehaven flood action group	GBR	No		x			x	x	x		x				45
Mytholmroyd flood group	GBR	No		x				x	x	x	x				46
Hebden Royd flood action group	GBR	No		x		x	x	x	x	x	x				47
Purley flood Defence group	GBR	No			x			x	x		x				48
**Cockermouth flood action group**	GBR	Yes	x	x			x	x	x	x	x				49
Todmorden flood group	GBR	No		x				x	x	x	x				50
Tadcaster flood action group	GBR	No			x			x	x		x				51
Eye on Calderdale	GBR	No			x			x	x			x			52
Hebden Royd flood action group	GBR	No		x				x	x	x	x				53
Whalley & Billington ‐ flood action group	GBR	No		x			x	x		x	x				54
Bodenham flood protection group	GBR	No			x		x	x			x				55
Charlton flood group	GBR	No	x	x			x				x				56
Brompton flood prevention group	GBR	No		x			x	x			x				57
Carlisle flood action group	GBR	Yes	x				x				x				58
Glastonbury Northload Bridge residents flood risk action group	GBR	Yes	x			x					x				59
National Flood Forum	GBR	No		x		x		x	x	x			x		60
Morpeth flood action group	GBR	No		x			x				x				61
Skibbereen flood committee (SFC)	IRL	Yes		x			x				x				62
Clontarf resident's association	IRL	Yes		x			x				x				63
West Virginia flood relief community connection	USA	No		x						x		x			64
RainReady	USA	No			x	x	x	x	x	x			x		65
2016 flood of cedar rapids	USA	No		x					x	x	x				66
The gross gatherings	USA	No		x			x	x	x		x				67
Hands on Nashville: Waterway cleanup, restoration and emergency response	USA	No		x			x		x	x	x				68
Rain ready Floodlothian Midlothian	USA	No		x	x	x	x	x			x				69
Flood forum for Connellsville City	USA	No		x						x	x				70

x, criteria satisfied; Int.,international. Country codes refer to ISO 3166‐1 Alpha‐3 standard; Selected study sites are highlighted in bold.

**Table A3:** (Online) Sources for BUI screening

Reference ID	Source
1	https://www.facebook.com/ulupnatobarmahfloodwatch/
2	https://www.facebook.com/Numurkah-Flood-Action-Group-521473864717743/
3	http://www.tt.com/politik/landespolitik/11126215-91/hohe-wellen-um-hochwasserschutz.csp
4	http://www.hochwasserschutz-eferdinger-becken.at
5	www.initiative-aist.at
6	http://www.graz.at/cms/beitrag/10085918/422037/
7	http://www.hochwasser-20.com
8	https://fraunberghochwasser.at/de/
9	https://www.teamoesterreich.at
10	http://www.siz.cc/weissenkirchen_w_/diesiz
11	http://www.luzernerbauern.ch/interessenvertretung/ig-reuss.html#medienspiegel
12	http://www.flut.arche-nova.org/de
13	http://www.hochwasserschutz-elbe-mulde.de/index.asp?Link=Der%20Verein
14	http://www.hwng-braubach.de
15	http://hwsi.de
16	http://www.rettet-das-selketal.de
17	http://nr-feldmann.de/D-HWS/D-Einfuehrung.html
18	http://hochwasserschutz-hexental.de
19	http://www.flutpolder-schwenningen-tapfheim.de/zusammenschluss-der-interessengemein-schaften-kontra-polder-an-der-oberen-donau/
20	http://www.bi-uebigau.de
21	http://www.dzonline.de/Duelmen/2013/07/Nur-der-erste-Schritt-ist-getan-Initiative-Hochwasserschutz-Buldern-moechte-Eventualitaeten-simulieren
22	http://www.elbe-aken.de
23	http://www.bi-bb.de
24	https://www.tag24.de/nachrichten/jahnsdorf-einwohner-kaempfen-fuer-rueckhalte-becken-67962
25	http://www.hochwasserschutz-kobern.de
26	http://www.bhn2013.de
27	http://www.bi-hochwasser-erfurt.de/index.htm
28	http://isarsiedlung.blogspot.co.at
29	http://www.bihn-altrip.de
30	http://www.schlichem.de
31	http://www.flutpolder-schwenningen-tapfheim.de
32	http://wertingen.blogspot.co.at/2016/08/burgerinitiative-rettet-das-donauried.html
33	Mees H.L.P., Driessen P.P., Runhaar H.A.C (2014) Legitimate adaptive flood risk governance beyond the dikes: the case of Hamburg, Helsinki and Rotterdam. Regional Environmental Change, 14(2), 671–682.
34	Zehetmair, S. (2012) Zur Kommunikation von Risiken. Eine Studie über soziale Systeme im Hochwasserrisikomanagement. Springer, Wiesbaden.
35	http://www.hochwasser.de/buergerinitiative-hochwasser-koeln-startseite.html
36	http://www.b-b-e.de/fileadmin/inhalte/aktuelles/2013/10/NL22_DRK_Definition.pdf
37	http://www.floodaction.org.uk/blog/
38	http://www.isbournecatchment.org.uk
39	http://www.flagsomerset.org.uk
40	http://www.lancsfloodappeal.org.uk
41	http://www.floodalleviation.co.uk/
42	http://edenfloodvolunteers.org
43	http://www.sedgeberrow.com/floodgroup/index.html
44	http://cerg.org.uk
45	http://www.stonehavenfloodaction.org
46	http://eyeoncalderdale.com/community/mytholmroyd-flood-group
47	http://eyeoncalderdale.com/community/hebden-bridge-action-group
48	http://purleyflooddefence.org.uk
49	Thaler T., Levin‐Keitel M. (2016) Multi‐level stakeholder engagement in flood risk management–A question of roles and power: Lessons from England. Environmental Science & Policy, 55, 292–301.
50	http://eyeoncalderdale.com/community/Todmorden-Flood-Group
51	http://tadcasterflood.org
52	http://eyeoncalderdale.com
53	http://eyeoncalderdale.com/community/hebden-bridge-action-group
54	https://whalleyandbillingtonfloodactiongroup.wordpress.com
55	http://www.bodenhamparish.org.uk/bfpg-home.asp
56	http://www.nationalfloodforum.org.uk/flood-risk-community-groups/community-flood-plans/
57	http://bromptonfloodpreventiongroup.btck.co.uk
58	http://www.carlislefloodaction.org.uk
59	https://www.facebook.com/Glastonbury-Northload-Bridge-Residents-Flood-Risk-Action-Group-222269481446298/
60	http://www.nationalfloodforum.org.uk/
61	Thaler T., Levin‐Keitel M. (2016) Multi‐level stakeholder engagement in flood risk management–A question of roles and power: Lessons from England. Environmental Science & Policy, 55, 292–301.
62	Clarke D., Murphy C. (2016) Societal transformation and adaptation necessary to manage dynamics in flood hazard and risk mitigation. WP2: Ireland Country Report.
63	Clarke D., Murphy C. (2016) Societal transformation and adaptation necessary to manage dynamics in flood hazard and risk mitigation. WP2: Ireland Country Report.
64	https://www.facebook.com/groups/565543666940725/
65	http://rainready.org
66	https://www.facebook.com/2016floodofCedarRapids/
67	http://www.heatofthemoment.org/features/flood/
68	https://www.hon.org/waterways
69	http://www.villageofmidlothian.net/335/RainReady
70	https://www.facebook.com/groups/1790878427817243/permalink/1794310630807356/

Online sources accessed in March 2017.
